# Form-Meaning Relations in Russian Confirmative and Surprise Declarative Questions

**DOI:** 10.1177/00238309251314862

**Published:** 2025-03-29

**Authors:** Andrei Munteanu, Angelika Kiss

**Affiliations:** University of Toronto, Canada

**Keywords:** Declarative questions, belief change, commitment, Russian, intonation, principal component analysis

## Abstract

Declarative questions (DQs) are declarative sentences used as questions. As declaratives, they differ from information-seeking polar questions (ISQs) in their syntax, and as biased questions, they differ from polar questions because they can convey various epistemic stances: a request for confirmation, surprise, or incredulity. Most studies on their intonation typically compare just one subtype to ISQs. In this paper, we present a production study where participants pronounced ISQs, confirmative and surprise DQs, and assertions in Russian. We analyzed the pitch and duration of the target utterances, as these prosodic cues proved to be important in the formal markedness of various biased question types across languages. A principal component analysis (PCA) on the pitch contours shows that DQs bear the same rise-fall contour as ISQs, but its peak falls on the stressed syllable of the last word of the sentence instead of the verb. The intonation of surprise DQs differs from that of confirmative ones in that they also exhibit a slight peak on the subject. Pitch alone is thus enough to distinguish the four utterance types tested. The PCA analysis was also used to identify higher-level trends in the data (principal components), two of which appear to correspond to core semantic properties, namely belief change and commitment. In addition to intonation, speaker commitment also correlates with utterance duration.

## 1 Introduction

A declarative question is a declarative sentence uttered with a question-like intonation (Abeillé et al., 2014; Farkas & Bruce, 2010; Gunlogson, 2003; Kiss, 2024; Krifka, 2015; Poschmann, 2008; Prieto, 2015; Trotzke, 2023). Consider the context in (1) and A’s possible utterances. By (1a), A could express that she has just learned the fact that B missed his train. This is done by uttering a declarative sentence in a way that conveys an assertion, which is the most typical function of declarative. By (1b), A would express a question and would expect an answer from B. This is conveyed by an interrogative sentence pronounced in a way that conveys the speech act of an information-seeking polar question (ISQ). (1c), on the other hand, is an example of what is called a declarative question: a declarative sentence, which in English is seen from its word order, pronounced in a way that makes it a questioning speech act, which in English is said to involve an utterance-final rise. This question also conveys a bias: the speaker has a non-neutral epistemic stance on the issue raised by the question (Gunlogson, 2003, 2008; Krifka, 2015; Trotzke, 2023); in this particular case, an inclination toward the positive answer. This bias is one that has been recently acquired based on some contextually available piece of evidence that the speaker has just encountered (Kiss, 2024). Due to this property, declarative questions (DQs) are considered biased questions.

(1) Context: A sees that B is standing alone on the platform at the train station at 8.05 am, and A knows that B takes the 8.00 am train every day. A to B:
  a. You missed the train. (Declarative sentence, assertive function)
  b. Did you miss the train? (Interrogative sentence, questioning function)
  c. You missed the train? (Declarative sentence, questioning function)

It is important, for our purposes, to distinguish declarative and interrogative sentences as syntactic objects on the one hand, from assertions and questions as speech acts, on the other. While declaratives are canonically associated with assertions, and interrogatives, with questions, there exist marked or non-canonical utterance types in which these associations are violated. Rhetorical questions are an example of such a case: they are interrogatives which show various properties of assertions (Farkas, 2020; Han, 2002; Kiss & Lo, 2021), and so are DQs, in which case a declarative sentence conveys a question.

In English, interrogatives are differentiated from declaratives by word order, but in Russian, they are differentiated from declaratives by intonation. That is, the same sentence can be used as a declarative or an interrogative, depending on its intonation. Bryzgunova (1977) characterizes assertions by a low tone on the last stressed syllable of the utterance, marked by ^1^ as shown in (2a), and polar interrogatives that convey ISQs, by a rise-fall contour on the stressed syllable of the verb, marked by ^3^, as shown in (2b).

(2) a. Opazdál na pó^1^jezd.
   was. late on train
   ‘He missed the train’                                (assertion)
  b. Opazdál^3^ na pójezd?
   was. late on train
   “Did he miss the train?”                        (information-seeking polar question)

In the second utterance of (3), an example from Bryzgunova (1977), which is what we call a declarative question, we see the same intonational contour as in ISQs (Meyer & Mleinek, 2006), but located where assertions have their characteristic low tone, the stressed syllable of the last word.

(3) Pochemú on ne prijéxal? Opazdál na pó^3^jezd?
   why  he not came was.late on train
   “Why did he not come? He missed the train?”                     (Bryzgunova, 1977, p. 136)

As such, the intonation of (3) is different from that of both assertions, like (2a), and from ISQs like (2b). It also differs from both utterance types in that it expresses a bias, namely that the speaker believes it to be likely that the addressee missed the train, based on some contextually available piece of evidence (i.e., seeing the addressee on the platform). All these properties are discussed in more detail later: see section 3.1 on Russian intonation, section 3.2 for diagnostic tests that show that DQs like the second utterance in (3) are indeed declarative sentences, and section 2 for an overview of the semantic properties.

DQs have been well-studied in English in terms of both meaning and form (Bartels, 1999; Farkas & Roelofsen, 2017; Geluykens, 1988; Gunlogson, 2003, 2008; Jeong, 2018; Malamud & Stephenson, 2015; Poschmann, 2008; Rudin, 2022). While the meaning and functions of this question type may be similar across languages, their form may vary from language to language. However, few studies are available on their form in languages other than English. This growing body of literature includes studies on Dutch (Beun, 1990), German (Petrone & Niebuhr, 2014), Bari Italian (Grice & Savino, 2003), Manchego Peninsular Spanish (Henriksen, 2012), and Hungarian (Gyuris, 2019; Mády et al., 2023). As for Russian, we are only aware of Makarova’s (2000) study, which compared the form of one-word DQs to string-identical ISQs.

Furthermore, DQs have various subtypes, which differ in the epistemic stance they convey (Kiss, 2024; Poschmann, 2008; Rudin, 2022): they can express a request for confirmation, surprise, or even incredulity. Differences in the speaker’s epistemic stance have been reported to be marked in prosody (Bartels, 1999; González et al., 2017; Henriksen, 2012; Orrico & D’Imperio, 2020; Prieto & Borràs-Comes, 2018; Vanrell et al., 2017). Yet, there are but a few studies that compare various subtypes of DQs to each other in terms of their formal markedness (see Bartels, 1999; Michelas et al., 2013; or Poschmann, 2008). Our paper thus addresses a gap in the literature in that it compares confirmative and surprise DQs, and in that it looks at Russian, in which, to our knowledge, the prosody of DQs has not yet been investigated in the production of multi-word utterances.

The pitch measurements collected in the study were analyzed by principal component analysis (PCA), which allows for the statistical analysis of high-dimensional and correlated data, such as intonation contours. The results of the study reveal that the four utterance types tested can be differentiated with just two principal components, which appear to correspond to two independently justified semantic properties, to be introduced in the following section: belief change and speaker commitment.

## 2 Declarative questions

DQs can convey various speaker attitudes. Consider example (4) below. In this context, Robin is exposed to contextual evidence for the fact that it is raining, namely the wet raincoat and boots of her colleague. Robin is thus biased toward the fact that it is raining, but for whatever reason, she needs her colleague’s acknowledgment to commit to the proposition that “it is raining.” A context in which the speaker is biased and wants to get confirmation from the addressee is one of the typical contexts in which DQs are used felicitously.^1^ Following Poschmann (2008), we refer to such DQs as confirmative declarative questions.

(4) Confirmative Declarative Question
   Robin is sitting in a windowless computer room when her colleague enters. The newcomer is wearing a wet raincoat and boots.
Robin says:
   a. It’s raining?                        (adapted from Gunlogson, 2003, p. 128b)

Another typical context of DQs is shown in (5). Robin in this case is surprised: the declarative question here echoes her colleague’s utterance and expresses that rainy weather is something Robin did not expect. We refer to such DQs as surprise declarative questions.

(5) Surprise Declarative Question
   Robin talks to her colleague on the phone. They are in the same building but only Robin’s colleague’s room has windows; Robin has no access to the current weather.
   a. Robin’s colleague: It’s raining.
   b. Robin: (What?) It’s raining?

The present paper focuses on these two subtypes, confirmative and surprise DQs. We do not discuss incredulous DQs, the third main type found in the literature, by which a speaker can convey incredulity (see Farkas & Roelofsen, 2017 or Kiss, 2024). We continue this section by describing key meaning components of DQs building on the works of Büring and Gunlogson (2000), Farkas and Roelofsen (2017), and Kiss (2024), and by clarifying how DQs relate to what are called “rising declaratives” in the literature.

### 2.1 The meaning of confirmative and surprise DQs

We assume that an ISQ like *Is it raining?* denotes the set of its possible answers (Hamblin, 1973): {‘it is raining,’ ‘it is not raining’}. Following Farkas and Roelofsen (2017), we assign the same denotation to DQs such as (4) and (5).^2^ DQs thus are assumed to have the same denotation as ISQs, (which are also commonly called “yes/no questions” or “genuine” or “canonical” polar questions), but the two question types are not always interchangeable across pragmatic contexts. As Gunlogson (2003) points out, only ISQs are felicitously used in an unbiased context such as a tax form. The question in (6a) is felicitous in this context, because it is a syntactically interrogative sentence, which can convey an ISQ (Farkas, 2020; Farkas & Bruce, 2010; Sudo, 2013), but (6b), which is a declarative question, is not felicitous. Gunlogson argues that the unavailability of DQs in contexts where the speaker (or author) is supposed to be unbiased or impartial shows that DQs convey bias.

(6) [on a tax form]
   a. During the tax year, did you receive a distribution from a foreign trust?
   b. #During the tax year, you received a distribution from a foreign trust?  (Gunlogson, 2003, p. 14)

Confirmative DQs like (4) and surprise DQs like (5) are similar in that the speaker is not completely ignorant with respect to the truth of the proposition ‘it is raining’, which is the sentence radical (the “descriptive content” of the utterance, see Stenius, 1967), marked as *p*. But they differ in the kind of bias they convey. By a confirmative declarative question like (4), the speaker signals that they originally had a neutral stance toward *p*, but due to some contextually salient piece of evidence, they are now biased toward *p*; in other words, the evidence made the speaker think that *p* is likely true. In other words, the speaker signals that they consider ‘it is raining’ more likely to be true than ‘it is not raining’. However, this is just a bias, as the speaker does not yet fully believe that this proposition is true. The discourse move by which a speaker publicly endorses a proposition is called commitment (Farkas & Bruce, 2010): when the speaker asks a confirmative declarative question, they express a bias toward the sentence radical but are not committed to it.^3^ In the case of surprise DQs like (5), the speaker signals that, even though prior to encountering contextual evidence for *p*, they believed *p* was more likely to be false than true, now they believe that *p* *is* true. A surprise declarative question like (5) thus commits the speaker to *p*. In sum, confirmative DQs convey a bias toward *p*, while surprise DQs commit the speaker to *p*, and both declarative question types also signal that this belief of the speaker has been acquired recently.

We compare the two declarative question types to ISQs and assertions, because they share properties with both. Confirmative DQs share with ISQs that the speaker is not (fully) committed. By asking an ISQ (*Is it raining?*) or a confirmative declarative question (*It’s raining?*), the speaker needs an answer from the addressee before they can commit to the proposition ‘it is raining’. Surprise DQs share with assertions that the speaker expresses commitment: in both cases, they commit to the truth of ‘it is raining’. On the other hand, DQs are different from both assertions and ISQs in that they do not signal a change in the speaker’s beliefs. Table 1 summarizes these properties of DQs, and how they compare to ISQs and assertions.

**Table 1.** Speaker commitment expressed by information-seeking questions (ISQs), confirmative and surprise declarative questions (DQs), and assertions.

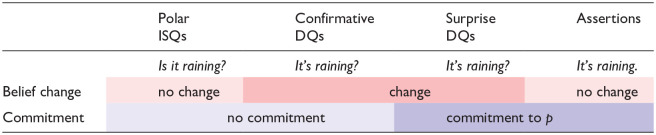

The sentence radical “it is raining” is marked as *p*.

Table 1 shows that DQs are distinguished from both ISQs and assertions because they express a change in the speaker’s belief about *p*; and it also shows that ISQs and confirmative DQs differ from surprise DQs and assertions in that the former do not commit the speaker to the truth of the sentence radical (*p*), while the latter do.

### 2.2 DQs and rising declaratives

Before concluding the description of DQs, an important note regarding terminology is made. DQs are often referred to as “rising declaratives” (Farkas & Roelofsen, 2017; Gunlogson, 2003, 2008; Heeren et al., 2015; Hirayama, 2022; Jeong, 2018; Rudin, 2022; Trinh & Crnič, 2011; Wakefield, 2014; Westera, 2018), and even though the two notions are similar, they do not cover the same range of utterance types. In English, rising declaratives encompass any utterance type in which a declarative sentence is associated with a final rising tune. Uptalk, an example of which is shown in (7), has been proposed by Gunlogson (2003) to belong to rising declaratives. Since the caller in (7) is making a statement by saying *I’m from Skokie?*, this utterance type does not belong to DQs, because it conveys the speech act of assertion, while DQs are meant to convey the speech act of questioning (Jeong, 2018).

(7) Radio station DJ: Good morning Susan. Where are you calling from?
   Caller: I’m from Skokie?                           (adapted from Gunlogson, 2003)

That is, we follow Jeong (2018) in separating what she calls inquisitive rising declaratives like (4) and (5), which convey questions, from assertive rising declaratives like (7), which convey assertions, and only the former type do we consider as DQs.

The term “rising declaratives” is not used here because across languages, this utterance type does not necessarily involve a single utterance-final rise. In Hungarian, DQs bear multiple rise-fall contours (Gyuris, 2019), identical to the interrogative contour (L*H-L%) that in a ISQ stretches over the entire utterance (Varga, 2010). In Catalan, a (confirmation-seeking) declarative question is marked by a steeper rise (¡H+L*) compared to the rise in ISQs (Vanrell et al., 2012). In Bari Italian, ISQs have a rising intonation but confirmative DQs have a falling one (Grice & Savino, 2003). Thus in Hungarian, DQs do not rise; in Catalan, both ISQs and DQs involve pronouncing the same string with a rising tune; and in Bari Italian, DQs end with a falling tune, so calling them rising declaratives is again unfortunate. In addition, Geluykens (1988) noted that a final rise is not even the most frequent intonation found in (British) English DQs. We thus prefer to use the term “declarative questions” (following Poschmann, 2008, and Prieto, 2015, among others) to the term “inquisitive rising declaratives” which only suits a particular subset of languages, namely those in which DQs involve an utterance-final rise.

## 3 Russian intonation

In this section, we present the intonation of Russian assertions and ISQs in Bryzgunova’s (1977) system, and the intonation of DQs. All claims are made with reference to sentences with a neutral word order and without a narrow focus on any of their constituents.

### 3.1 Declarative and polar interrogative sentences in Russian

In Russian, polar interrogatives can be formed both by intonation and by the clitic *li* (Bailyn, 2012; Comrie, 1984; Dryer, 2013; King, 1994; Korotkova, 2023; Meyer & Mleinek, 2006). Even though *li* is used obligatorily in subordinate questions, it only optionally marks matrix interrogatives, which are instead primarily marked by intonation (Meyer & Mleinek, 2006; Svetozarova, 1998). As *li* is somewhat archaic and marked, it is used in what Timberlake (2004) calls “written (or bookish oral) Russian.” A minimal pair is shown in (8).

(8) a. Ivan   SMOtrit televizor?
    Ivan-NOM watches TV
    ‘Is Ivan watching TV?’
  b. Smotrit li Ivan televizor?
    watches Q Ivan TV
    ‘Is Ivan watching TV/’                                    (Bailyn, 2012, p. 82)

In this paper, we only consider the unmarked way of forming interrogatives conveying ISQs, which is by means of intonation. In (8a), the intonational peak falls on the verb, *smotrit* ‘watches’, which is also the “locus of the semantic operation” in the sense that the denotation of this ISQ is generated by the positive and negated form of the verb: {‘Ivan watches television,’ ‘Ivan does not watch television’}.

As for the form of the intonation contour, we employ the system from Bryzgunova (1977), which distinguishes seven “basic types of intonational contours” (pp. 120–121), abbreviated here as IC. As Kachkovskaia et al. (2020) describe it, in Bryzgunova’s (1977) system, the basic segmental unit is the intonation phrase, within which there is one main word that bears the intonation contour (Timberlake, 2004).

Each intonation contour may be compatible with various sentence types or utterance types. For our purposes, the relevant contours are those that appear in declarative and interrogative sentences. IC-1 and IC-3, marked in example (9) by the superscripts ^1^ and ^3^ on the stressed syllables, distinguish declarative and interrogative sentences conveying assertions and information questions, respectively (see also Bryzgunova, 1975).

(9) a. Máma varíla ú^1^zhin (assertion)
   mother cooked dinner
    “Mother cooked dinner.”
  b. Máma varí^3^la úzhin (ISQ)
    mother cooked dinner
    ‘Did Mother cook dinner?’

In (9a), we see a declarative sentence conveying an assertion with a falling sentence-final contour, IC-1, which, according to Kachkovskaia et al. (2020), is equivalent to L* in Odé’s (2008b) ToRI system. The locus of the intonational contour that characterizes assertions is the rightmost stressed syllable, that is, the stressed syllable of the last word, in this case, the first syllable of *úzhin* ‘dinner’ (see also Igarashi, 2006, and Svetozarova, 1998). (9b), on the other hand, is an interrogative sentence conveying an ISQ which is marked by a rise-fall intonation, the peak of which falls on the stressed syllable of the verb, *varíla* ‘cooked’ (Svetozarova, 1998). This intonation contour is Bryzgunova’s (1977) IC-3, which Odé (2008a) and Igarashi (2006) analyze as LH*L, a contour associated with ISQs (Odé, 2005, 2008a, but see Odé, 2008b, who analyzes it as H*L).^4^

According to Bryzgunova (1977), IC-3 is a contour that can be found in speech acts other than questions as well, such as requests and exclamations, see (10).

(10) a. Zakrój^3^te oknó!
    close    window
    ‘Close the window’                           (Bryzgunova, 1977)
   b. Ox i   grú^3^byj zhe ty!
    Oh and rude    ZHE you
    ‘Oh are you ever rude’                          (transcribed from Timberlake, 2004)

However, we do not evaluate this claim as regardless of whether it holds, it does not directly affect our research.

IC-1 and IC-3 are described in (11) by Bryzgunova (1977) as follows:

(11) a. IC-1:
    “The precontour is pronounced with a middle tone which lowers on the center to varying degrees, and remains lower on the tail” (pp. 18–19). “[IK-1 is used] when there is no discourse division in the sentence [O-Theme], emphasis or transformation of word order.” Bryzgunova (1977), as translated and cited by Bailyn (2012)
  b. IC-3:
    “The precontour is pronounced with a middle tone, which is followed by a sudden raise in the center of the syllable until the end of the syllable. The postcontour consists of a tone which lowers below the middle tone of the precontour.” (Bryzgunova, 1977, p. 38, own summary)

In addition to the differences in their shapes, IC-1 and IC-3 are also associated with different parts of the sentence in assertions and ISQs. IC-1 (noted in example (12) as IK-1) falls at the end of a sentence, which, according to Bailyn (2012), is captured by the Nuclear Stress Rule proposed by Rochemont (1986) (who attributes it to Chomsky & Halle, 1968).

(12) Nuclear Stress Rule
  Assign IK-1 to the rightmost lexical category in S.                            (Rochemont, 1986)

**Figure 1.**
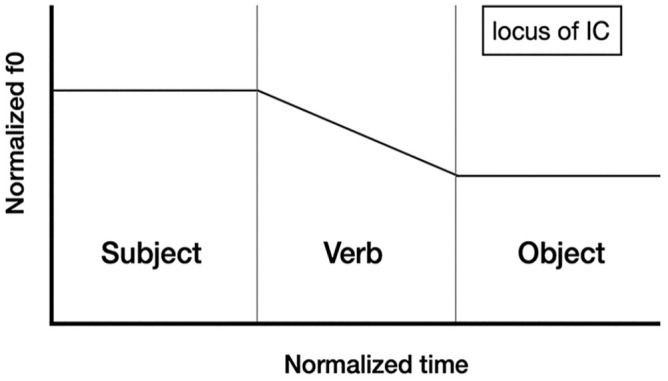
The locus of the falling intonation contour (IC-1) in declarative sentences with basic word order conveying assertions.

**Figure 2.**
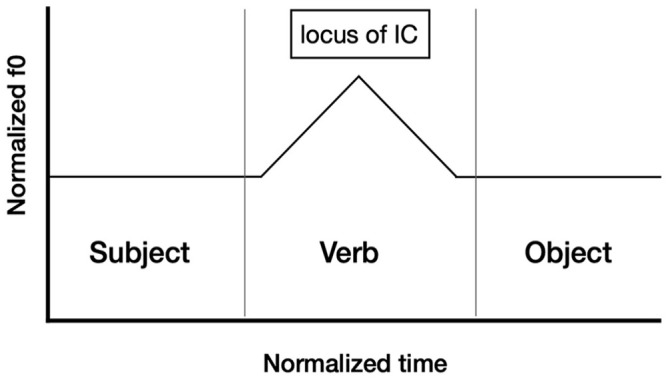
The locus of the rise-fall intonation contour (IC-3) in polar interrogative sentences with a basic word order conveying ISQs.

IC-3, on the other hand, falls on the stressed syllable of the verb. The two contours are represented schematically in Figures 1 and 2.

Since IC-3 is also used to mark focus intonation (Bailyn, 2012), associating it with the predicate of an ISQ creates two alternatives, one in which the predicate is true of the subject, and another one in which it is not, which is taken to be the denotation of ISQs (see section 2.1).^5^

These two intonation contours are strongly associated with assertions and questions, respectively, as they are distinguishable even in utterances that consist of no more than one word (Makarova & Petrushin, 2007).^6^

### 3.2 DQs in Russian

Examples of DQs are mentioned in the descriptive work of Bryzgunova (1977), even if they are not called as such. An example of a confirmative declarative question has been shown in (3), and repeated here in (13). The speaker asks the addressee about why a certain person did not arrive, and, immediately after, makes a guess in the form of a declarative question, revealing their bias toward the proposition that that person missed the train.

(13) Pochemú on ne prijéxal? Opazdál na pó3jezd?
    why  he not came was.late on train
    ‘Why did he not come? He missed the train?’                    (Bryzgunova, 1977, p. 136)

As it is shown by the superscript, Bryzgunova characterizes this question (*Opazdál na pójezd?*) by IC-3, the same contour type that marks ISQs, although in this case, the peak (marked by the superscript) does not appear on the stressed syllable of the verb but on the stressed syllable of the last word of the utterance. As such, the question *Opazdál na pójezd?* cannot be an ISQ; we propose that it is a declarative question.

While the second utterance of (13) is clearly a question, whether it is syntactically a declarative sentence is not obvious. Therefore, we perform some tests that set apart declarative sentences from interrogatives. First, we note that the question particle *li* can only occur in questions where IC-3 appears on the stressed syllable of the verb, that is, in syntactically interrogative sentences; they cannot be added to a question where IC-3 is located on the last stressed syllable of the sentence.

(14) a. Opazdál^3^ (li) na pójezd? (ISQ)
    was.late Q on train
    ‘Did he miss the train?’
  b. Pochemú on ne prijéxal? Opazdál (*li) na pó^3^jezd? (DQ)
    why   he not came  was.late Q  on train
    ‘Why did he not come? He missed the train?’

Second, Abeillé et al. (2014) note that the phrase “that is the question” can only refer to ISQs conveyed by interrogatives, not to DQs. This holds in French and English, as well as in Russian, where the contrast is shown in the locus of the rise-fall intonation marked by ^3^.

(15) a. Est-ce qu’il a pris le train? Telle est la question. (ISQ)
    ‘Did he catch the train? That’s the question.’
  b. Il a pris le train? #Telle est la question. (DQ)
    ‘He caught the train? #That’s the question’.
  c. On uspél^3^  na pójezd? Vot v chem voprós. (ISQ)
    he made.it on train  here in what question
    ‘Did he catch the train? That’s the question.’
  d. On uspél  na pó^3^jezd? #Vot v chem voprós. (DQ)
    he made.it on train here in what question
    ‘He caught the train? #That’s the question.’

Third, following Abeillé et al. (2014), we use the diagnostic of negative polarity items (NPIs) such as хоть когда-нибудь (*xot’ kogdá nibúd’*) ‘ever.’ In the absence of a negation word, an NPI can only occur in interrogative sentences but not in declarative ones. In examples (16)–(18), we cited the original French examples of Abeillé et al. (2014) with their English translation and provided the Russian equivalents.

(16) Information-seeking questions
  a. Ces jeunes gens avaient-ils **jamais** lu un roman classique? (French) these young people have-they ever  read a novel classical
  b. Éti  yúnoshi   **xot’ kogdá-nibúd’** chitá3li klassícheskij román? (Russian) these young.people ever       read classical  novel
  c. Had these young people **ever** read a classical novel?
(17) Declaratives (assertive function)
  a. Ces jeunes gens avaient (*jamais) lu un roman classique. (French) these young people have  ever   read a novel classical
  b. Éti yúnoshi (*xot’ kogdá-nibúd’) chitá1li klassícheskij román. (Russian) these young.people ever      read classical  novel
  c. These young people have (*ever) read a classical novel.
(18) Declaratives (question function)
  a. Ces jeunes gens avaient (*jamais) lu un roman classique? (French) these young people have  ever   read a novel classical
  b. Éti yúnoshi   (*xot’ kogdá-nibúd’) chitáli klassícheskij román3? (Russian) these young.people ever        read classical  novel
  c. These young people have (*ever) read a classical novel?

The following pattern can be seen: interrogative sentences that convey ISQs can host the NPI *ever* and its Russian and French counterparts (16), but they cannot appear in declarative sentences, whether they convey an assertion (17) or a declarative question (18). Thus, whether a question like the second utterance in (13) is a declarative or interrogative sentence depends on whether it can host an NPI. As we show below in (19), хоть когда нибудь ‘ever’ is only licensed in the sentence if the rise-fall contour (IC-3) is on the stressed syllable of the verb, as in (19a).^7^

(19) a. Ty (xot’ kogdá-nibúd’) opáz^3^dyval na pójezd? (ISQ)
    you ever      were.late  on train
    ‘Did you ever miss the train?’
  b. Ty (*xot’ kogdá-nibúd’) opázdyval na pó^3^jezd? (DQ)
    you ever       were.late  on train
    Intended: ‘Did you ever miss the train?’

While there are further tests that can be applied, we refer the reader to the works of Abeillé et al. (2014) and Gyuris (2019) who used them to distinguish declarative and interrogative sentences from each other in French and Hungarian, respectively. The tests presented in this section show that SVO sentences where IC-3 is not on the verb but on the object behave as declaratives do, and so despite their question-like intonation, we conclude that such utterances are indeed DQs.

Based on Bryzgunova’s (1977) intonation system, there are two prosodic parameters to consider in Russian utterances: the shape of the contour and its alignment within the utterance. We argue that Russian DQs exhibit a mix of the properties of Bryzgunova’s (1977) IC-1, a low tune aligned to the rightmost stressed syllable in the utterance that marks assertions, and IC-3, a rise-fall contour aligned to the stressed syllable of the verb that is characteristic of questions. Specifically, the declarative component is provided by the alignment of the peak, in that the peak appears in the position where IC-1 would appear in a declarative sentence, which is the rightmost stressed syllable. And the interrogative component is contributed by the shape of the contour, exhibiting IC-3. In sum, DQs in Russian bear an intonation that is different from both assertions and ISQs.

In other words, based on Bryzgunova’s descriptive analysis, we hypothesize that in Russian DQs, IC-3, the intonation contour characteristic of questions, is aligned to the position of the key contour of an unmarked declarative sentence.

### 3.3 Previous work on Russian DQs

Surprise DQs have been compared to ISQs in production by Makarova (2000) who elicited three-syllable-long CVCVCV words (for example *Marína*) in the two relevant question readings.^8^ The information-seeking question condition was represented by a single question mark at the end of the utterance (i.e., *Marína?*), and the surprise question reading, with two question marks (i.e., *Marína??*). Since the target stimuli were all single-word utterances, and not sentences, the only locus of any intonation contour would be the same syllable, namely the stressed one (*ri* in *Marina*).

Although, based on our reasoning so far, the prediction would be that the two utterance types would sound the same, Makarova (2000) found that ISQs and surprise DQs differ in a number of prosodic properties. First, the stressed vowel (*i* in *Marína*) appears longer in surprise DQs. Second, the onset of the stressed syllable, which is the locus of IC-3, is lower in pitch and the pitch peak of the IC-3 contour is higher in surprise DQs than ISQs. Third, the rise of IC-3 in surprise DQs appears to be delayed compared to ISQs, and as a result, the peak is realized later in surprise DQs. And finally, she found that when said as a surprised utterance, the duration of the word was significantly longer than when it was pronounced as an ISQ.

While Makarova’s (2000) experiment is valuable given the scarceness of empirical research on this topic, especially in languages other than English, it only compares ISQs to (surprise) DQs, but does not inform us about differences between different types of DQs.

Looking at empirical data when studying biased questions is crucial, as native speakers’ intuitions about their form cannot be fully trusted. One way of determining the prosodic properties of the two declarative question types in Russian is by means of experiments; the details of such an experiment are presented in the next section.

## 4 Production experiment

The production task experiment was designed and hosted on the online platform *Gorilla* (Anwyl-Irvine et al., 2020). All recordings were conducted on the participants’ own hardware without the direct involvement of the researchers. This was not deemed an issue, as recent studies have found that, at least when it comes to measuring F0, recording quality in online experiments is comparable to those performed in the lab (Ge et al., 2021; Python et al., 2023).

During the experiment, participants were tasked with pronouncing a target sentence in Russian. Each target sentence appeared in all four conditions: ISQs, confirmative DQs, surprise DQs, and assertions. Each condition was elicited by a preceding context and an interlocutor utterance. The experiment was designed to test the effect of utterance type on two prosodic features of the target sentence, F0 and duration.

### 4.1 Predictions

**Figure 3.**
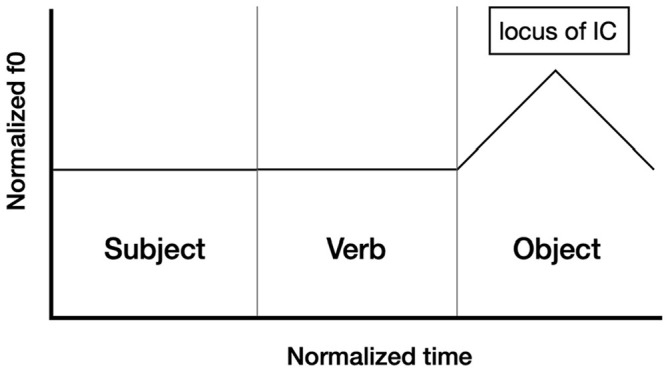
The schematic representation of the intonation of a declarative question on a Subject-Verb-Object string: IC-3 appears in the locus of IC-1.

Following Bryzgunova (1977), we predict that ISQs display a rise-fall intonation contour with the peak aligned to the verb (see Figure 2). We predict DQs to also display rise-fall contour, but that the peak will be instead aligned with the rightmost stressed syllable in the utterance, as illustrated in Figure 3 (for this experiment, corresponding to the stressed syllable of the object). Assertions are predicted to display falling intonation throughout the sentence.

Previous literature on Russian prosody does not address the difference between confirmative and surprise DQs. However, there is still reason to believe that the contrast is signaled prosodically, as prosody is known to be crucial in the expression of various kinds of epistemic stance, such as bias, commitment or surprise, both in Russian (Makarova, 1999, 2000; Svetozarova, 1998), and across languages (Asu et al., 2023; Bartels, 1999; Celle & Pélissier, 2022; Grice & Savino, 2003; Henriksen, 2012; Korotkova, 2023; and Rett & Sturman, 2021; a.o.). Therefore, we expect that differences in speaker commitment are reflected by differences in prosodic marking between the two declarative question types, as confirmative DQs do not express commitment, but surprise DQs do (see Table 1).

Finally, in accordance with previous findings on commitment and utterance duration, we expect duration to distinguish utterance types depending on whether they express commitment. Previous studies have found that utterances that do not express commitment are generally shorter than those that do. Beyssade and Delais-Roussaire (2022) report that speakers produced rhetorical questions with a negative answer, which expresses commitment, at a lower articulation rate than string-identical information-seeking questions, which do not. Similarly, Braun et al. (2018) observed that interrogatives differ in duration in German depending on whether they convey an information-seeking or a rhetorical question (with a negative answer), and Dehé and Braun (2019) found the same for English. Similar tendencies have been observed in Mandarin, where, in addition to the difference in utterance duration, a difference in wh-word duration was found between rhetorical questions and information-seeking questions (Dehé et al., 2018; Lo & Kiss, 2020). Finally, recall that Makarova (2000), too, has found that surprised utterances had a longer duration than ones expressing ISQs. While the speaker's commitment expressed by assertions, surprise DQs, and rhetorical questions is different, ultimately, they all commit the speaker to a certain proposition. As such, we predict that utterance types expressing commitment, namely assertions and surprise DQs, will exhibit a longer duration than utterance types that do not, namely ISQs and confirmative DQs.

### 4.2 Method

#### 4.2.1 Participants

Recordings were collected from 20 Russian-speaking participants. Recruitment was conducted through the *Prolific* website (www.prolific.co). Due to impressionistically poor recording quality, data from two of the participants was excluded. In addition, two participants were accidentally recruited twice, for whom only the first recording was used. Thus, the experiment results comprise data from 16 participants.

Self-reported demographic information was collected in a pre-task questionnaire and is presented in Table 2. The majority of participants were female (*F* = 13, *M* = 3). At the time of the experiment, the mean age of participants was 36 (median 34, range 25–59). All participants reported Russia or the USSR as their place of birth. Perhaps because the Prolific service is not as popular in Russia, for all but one participant, the current place of residence lay outside of the country. However, all of them report having been raised monolingual and monocultural and having lived in their birthplace at least until they reached adulthood.

**Table 2.** Demographic information of experiment participants.

Code	Gender	Year of birth	Place of birth	Place of residence
sp01	F	1994	Tver, Russia	Helsinki, Finland
sp02	F	1962	Moscow, Russia	Tampa, USA
sp03	F	1994	Votkinsk, Russia	Izhevsk, Russia
sp04	F	1964	Russia	USA
sp05	F	1988	Norilsk, Russia	Frankfurt, Germany
sp06	F	1976	Moscow, Russia	Japan
sp07	F	1991	Russia	USA
sp08	M	1978	Russia	Vancouver, Canada
sp09	M	1996	Russia	Germany
sp10	F	1988	Russia	Ireland
sp11	F	1984	Russia	USA
sp12	F	1982	Russia	Spain
sp13	M	1998	Ishalino, Russia	Haage, Netherlands
sp14	F	1994	Moscow, Russia	London, UK
sp15	F	1986	Moscow, Russia	London, UK
sp16	F	1986	Russia	Portugal

#### 4.2.2 Stimuli

Each trial consisted of a context, an interlocutor utterance, and a target sentence. There were four utterance-type conditions: ISQs, confirmative DQs, surprise DQs, and assertions. A total of seven target sentences were tested in each of the conditions, for a total of 28 critical items. The experiment also included 24 filler items, which did not vary by condition. The stimuli are publicly available at https://osf.io/cdszb/.

##### 4.2.2.1 Context

Each trial began with a context presented orthographically, ranging from 1 to 5 sentences in length. Contexts were designed to elicit one of the four utterance types and were tailored to the corresponding target sentence.

To ensure that the contexts elicited the correct utterance type, a pre-test context rating task was designed, which tested the three question types: ISQs, confirmative DQs, and surprise DQs. Since the interpretation of assertions is straightforward, assertion contexts were not included in the pre-test. As an example, (20) shows the pre-test choices for the potential target sentence мама варила ужин “mom cooked dinner” following a context.

(20) a. Вы не знаете, варила ли мама ужин или нет.
    ‘You don’t know if (your) mother cooked dinner or not.’
  b. Вы думаете, что мама варила ужин, но вы не уверены.
    ‘You think that (your) mother cooked dinner, but you are not sure.’
  c. Вас удивило, что мама варила ужин.
    ‘You are surprised that (your) mother cooked dinner.’
  d. Вас удивило, что мама варила ужин, и вы этому рады.
    ‘You are surprised that (your) mother cooked dinner, and you are happy about it.’
  e. Вас удивило, что мама варила ужин, и вы этому не рады.
    ‘You are surprised that (your) mother cooked dinner, but you are not happy about it.’

There were five options in the questionnaire because a surprise declarative question interpretation was tested for three levels of emotional valence. The three options all indicated that the speaker was surprised by the target sentence but varied in the speaker’s emotional response to the surprise: neutral (20c), positive (20d), and negative (20e). Because the speaker’s emotional response was not included into the production task itself, it will not be discussed further in this paper. All three options were collapsed into a single surprise declarative question response.

Thus if, upon reading the context, participants chose (20a), the context was scored as prompting an information-seeking question interpretation. If (20b) was chosen, the context was scored as prompting a confirmative declarative question interpretation. Finally, if any of (20c)–(20e) was chosen, the context was scored as prompting a surprise declarative question interpretation.

The pre-test consisted of contexts for 11 potential target sentences, each of which appeared in three utterance type conditions, for a total of 33 trials. Contexts in the questionnaire were presented orthographically. The pre-test items were split into three questionnaires using a Latin square design and disseminated to speakers of Russian. The three pre-test questionnaires were completed by a total of 28 speakers. Fourteen speakers (50%) were self-reported Russian monoglots; eleven speakers (39.3%) reported Russian as their first language and fluency in another language; the remaining three speakers (10.7%) reported another language as their first language and fluency in Russian. Ten speakers (35.7%) reported Russia as their place of birth; the remaining speakers were born in one of the former Soviet Republics.

The seven target sentences that elicited the intended interpretation the most in the three conditions tested (ISQ, confirmative DQ, surprise DQ) were selected to be used in the production task. None of the target sentences in the pre-test elicited the intended interpretation from participants in all three conditions all of the time. For the seven target sentences selected for the experiment, the intended judgment was elicited more than 60% of the time (*M*: 70.9%, range: 60.7%–89.2%).

##### 4.2.2.2 Interlocutor utterance

In the production experiment, the context of each trial introduced an interlocutor. The context screen was followed by the interlocutor utterance, designed to elicit a response from the participant (i.e., the target sentence). The interlocutor utterance varied by trial. Refer to Table 3, for an example of context, interlocutor utterance, and target sentence and to the supplementary materials available online.

**Table 3.** Example of a context, interlocutor utterance, and target sentence for an item in the assertion condition.

	Context	Interlocutor utterance	Target sentence
Original (Russian)	Вы собрались всей компанией на ужин у вашей подруги Марины. Ваш опаздывающий друг Дима вам звонит.	Я задерживаюсь. Я что ни будь пропустил?	Пока ничего. Марина разлила ви′на.
English translation	‘You and several others are having dinner at your friend Marina’s place. Your friend Dima, who is running late, calls you.’	‘I’m running late. Did I miss anything?’	‘Nothing so far. *Marina poured the wines.*’

To increase immersion in the experiment, a recording of the interlocutor utterance was played to the participants at the same time as a transcription (in standard orthography) was on screen. Interlocutor utterances were recorded from four speakers of Russian, two for the female characters and two for the male characters. Each speaker recorded all utterances of the corresponding gender. For every item, the researchers manually selected the recording that sounded the most natural in the context given. All four speakers were born in Russia or in one of the former Soviet Republics and resided in Canada at the time of recording.

##### 4.2.2.3 Target sentence

Participants were tasked with producing the target sentence in response to the interlocutor utterance. The target sentence was presented orthographically on a separate screen. The target sentence consisted of a subject, verb, and object. The seven target sentences appeared in one of the four utterance-type conditions for a total of 28 critical items. The full list of target sentences, as well as transcriptions in the International Phonetic Alphabet (IPA) and translations, can be found in Table 4.

**Table 4.** Experiment target sentences.

Number	Target (orthography)	Target (IPA)	Translation
1	Мама варила ужин	mama var^j^ila uʐin	‘Mom cooked dinner’
2	Аня развязала узел	an^j^a razv^j^azala uz^j^el	‘Anna undid the knot’
3	Полина уронила вазу	pol^j^ina uron^j^ila vazu	‘Polina dropped the vase’
4	Лера завернула мыло	l^j^era zav^j^ernula milo	‘Lera wrapped the soap’
5	Яна разложила лыжи	jana razloʐila liʐi	‘Yana laid out the skis’
6	Марина разлила ви′на	mar^j^ina razl^j^ila v^j^ina	‘Marina poured the wines’
7	Мы жарим зёрна	mi ʐar^j^im z^j^orna	‘We are frying beans’

The target sentence was composed of the subject, the verb, and the object, always in that order. To ensure that a glottal pulse would be present throughout the utterance, voiceless segments were avoided in all positions except the initial segment of the utterance. In case their burst obscures the glottal pulse measurements, voiced stops were also avoided.

In addition to the 28 critical items, there were 24 filler items. The structure of filler trials mirrored that of critical trials. For filler items too, a context was followed by an interlocutor utterance and by a target sentence. As in the case of critical trials, some filler trials also shared a “target sentence”. However, unlike for critical items, the contexts for filler items did not elicit different utterance types. The prosodic and segmental properties of the filler items were not controlled. The fillers were included to obscure the purpose of the experiment and avoid invoking prescriptive notions about intonation and utterance type.

To ensure that issues at the start of recording did not affect the experimental results, a short buffer phrase preceded the target phrase. The buffer phrase varied by trial. Because Russian marks grammatical gender on past tense verbs, there were a few target sentences that had to be modified depending on the gender of the participant. All modifications occurred in the buffer or in filler items. Target sentences in critical trials did not reveal the gender of the speaker. Gender was solicited in a demographic questionnaire immediately preceding the experiment. For a full list of experimental items, see the supplementary materials.

#### 4.2.3 Procedure

The experiment was preceded by a consent form and a technical check of the recording and sound quality. Experiment trials began with the context presented orthographically. After 3 s, a button labeled “Продолжить” (Continue) appeared. Thus, the participants were required to take no less than 3 s reading the context but could take longer if they wished.

On the following screen, the interlocutor utterance was presented orthographically. After a 1.5 s delay, a recording of the interlocutor sentence began to play. The trial progressed to the target sentence automatically once the recording finished playing.

On the final screen, the target sentence was presented orthographically. After a 3 s delay, a record button appeared on screen, giving participants the option to record their response to the interlocutor utterance. The same button, with the label updated accordingly, stopped the recording. Upon recording, participants were allowed to move on to the following trial at their own pace.

**Figure 4.**
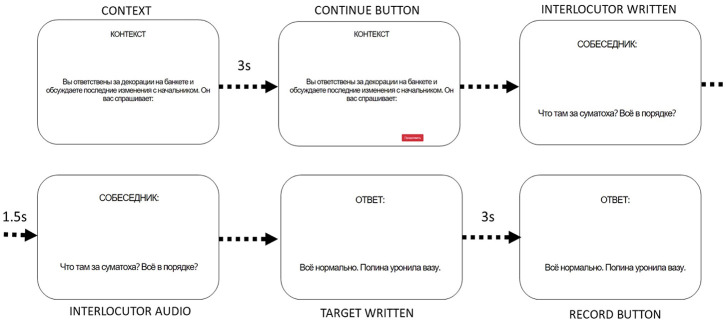
A schematic flowchart of the experimental procedure. Where progress to the next screen happened automatically, the time delay is given.

A schematized example of an experiment trial can be seen in Figure 4.

### 4.3 Results

Each word in the target sentences was annotated manually using Praat (Boersma & Weenink, 2019). Recording quality varied by participant but, at least for the 16 participants kept in the analysis, was impressionistically high. Sampling rate varied between 44,100 Hz and 48,000 Hz.

Pre-target buffers were not included in the annotation. Measurements of F0 were extracted at 20 evenly spaced points of each word using a script. Because the final measurement of the first word was the same as the initial measurement of the second and the final measurement of the second word was the same as the initial measurement of the third, a total of 58 unique F0 measurements were extracted per item.

**Figure 5.**
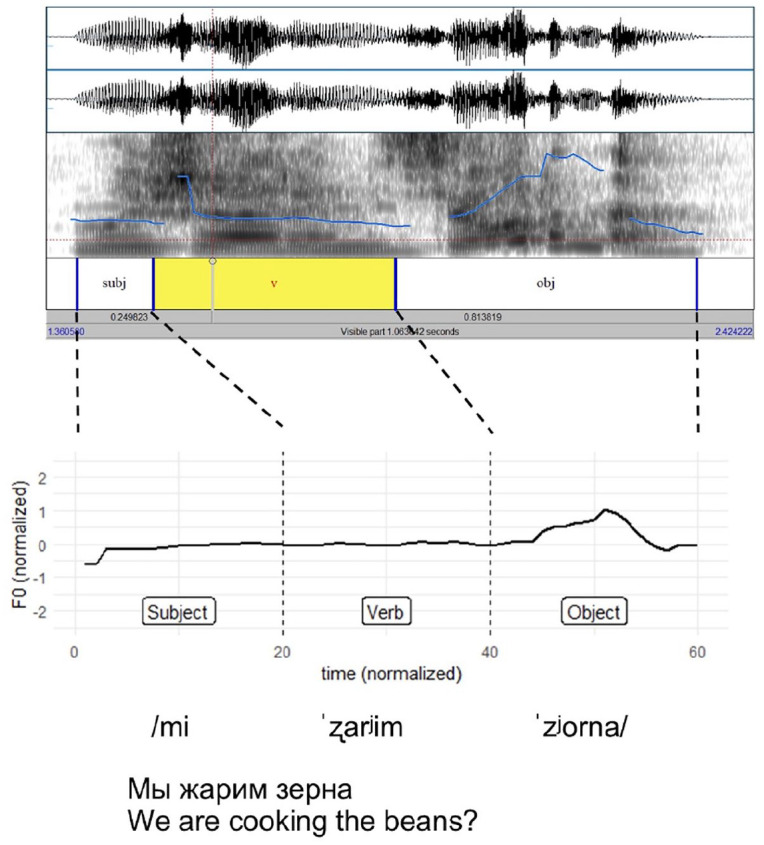
Sample Praat annotation and corresponding F0 contour after automated extraction.

All target sentences had the same Subject-Verb-Object structure. However, the length of these components varied between target sentences. Extracting a set number of measurements for each word, rather than the entire utterance, effectively controlled for variation in word length across target sentences. This is illustrated in Figure 5, where the subject /mi/ ‘we’ contributes as many F0 measurements as the object /z^j^orna/ ‘beans’. Normalizing the measurements in this way anchors prosodic features to the syntactic structure and allows for the comparison of F0 peaks and troughs across different target sentences.

Pitch measurements were normalized by speaker, allowing for between-speaker comparisons. Although extra care was taken to ensure that the target sentences would have a glottal pulse throughout the utterance, due to script error, noisy recording, or unexpected devoicing, the script was occasionally unable to extract pitch from the data. Missing F0 values (8.2%) were replaced with the average of adjacent measurements.

#### 4.3.1 Duration

**Figure 6.**
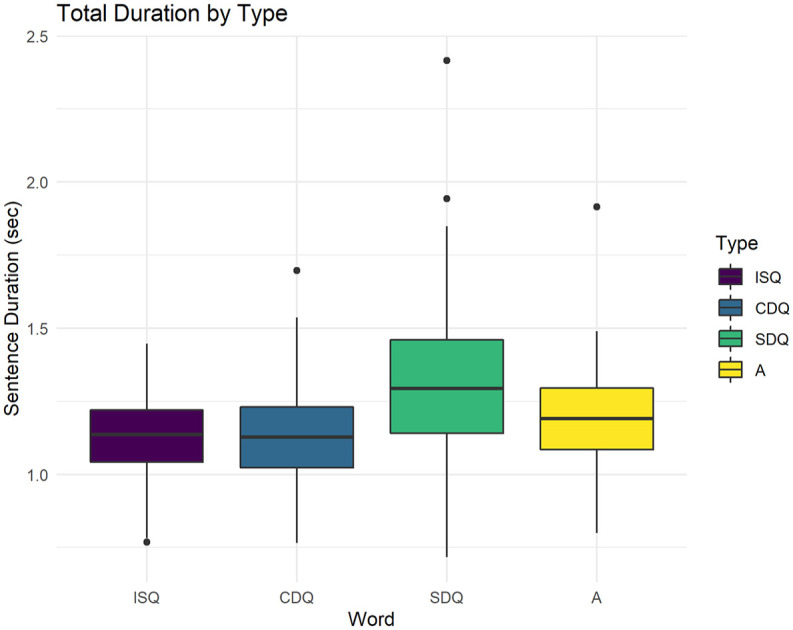
Boxplot of the target sentence duration by-utterance type. The box covers the second and third quartiles. The horizontal bar corresponds to the median value. Black dots are outliers.

This section discusses the correlation between utterance duration and utterance type. Total utterance duration had a mean of 1,190 ms and ranged from 720 ms to 2,420 ms. As can be seen in Figure 6, surprise DQs had the longest duration on average, with a mean of 1,310 ms, followed by assertions with a mean of 1,180 ms. There was no discernable difference in duration between confirmative DQs and ISQs, as both had an average duration of 1,130 ms.

To determine if utterance type had a significant effect on utterance duration, a mixed-effects model was run using the *lme4* package (Bates et al., 2015) in R (R Core Team, 2019). Utterance duration was set as the dependent variable, and utterance type (reference level: ISQ) was set as the independent variable. Participant and Item were included as random effects. By-utterance type slope per participant was also included in the model. The model formula can be seen in (21).

(21) duration ~ type + (type|participant) + (1|item)

Results for the target sentence duration mixed-effects model are presented in Table 5.

**Table 5.** Mixed-effects model results for duration.

	Estimate	*SE*	*t*
Intercept	1.1269915	0.0474773	23.737*
Utterance type CDQ	0.0004673	0.0191906	0.024
Utterance type SDQ	0.1846518	0.0421047	4.386*
Utterance type Assertion	0.0578322	0.0195469	2.959*

* *p* < 0.05.

The cut-off for statistical significance was set to an absolute *t*-value of 2. Thus, the results in Table 5 indicate a significant difference in duration between ISQs and surprise DQs and between ISQs and assertions. To see if duration differentiated between other sentence-type pairs, a post hoc test was run on the model interactions using the *phia* package (De Rosario Martinez et al., 2015) in R. Utterance type was set as the pairwise comparison. Results of the interaction test are given in Table 6. Note that *p*-values in Table 6 are adjusted using the Holm method.

**Table 6.** Post hoc interaction test results for duration.

	Value	*df*	*χ* ^2^	Pr(> χ^2^)
ISQ–CDQ	−0.000467	1	0.0006	0.98057
ISQ–SDQ	−0.184652	1	19.2329	< 0.0001***
ISQ–Assertion	−0.057832	1	8.77535	0.01236*
CDQ–SDQ	−0.184184	1	21.4858	< 0.0001***
CDQ–Assertion	−0.057365	1	8.2528	0.01236*
SDQ–Assertion	0.126820	1	6.4304	0.02244*

* *p* < 0.05; ***p < 0.001.

The results of the interaction test confirm the initial intuitions: the difference between ISQs and confirmative-DQs is not significant. All other comparisons are significant. Surprise DQs exhibit the longest duration, followed by assertions, followed by the remaining two utterance types.

It should be noted that the difference in utterance duration between utterance types likely manifests itself in several acoustic properties. Previous studies have shown that speech rate affects the likelihood of vowel neutralization and gestural overlap; moreover, changes in speech rate affect vowel duration to a greater extent than consonant duration (Gay, 1981; Rosner & Pickering, 1994). As such, we expect ISQs and confirmative DQs to have proportionally shorter vowel duration than assertions and surprise DQs. In addition, we expect the vowels in ISQs and confirmative DQs to exhibit some target undershoot, resulting in a smaller vowel space overall. Finally, we expect a greater degree of vowel-consonant coarticulation in the shorter ISQs and confirmative DQs. However, because these secondary effects are correlated with total utterance duration (i.e., speech rate), it makes little sense to test them independently for this project.

#### 4.3.2 F0

**Figure 7.**
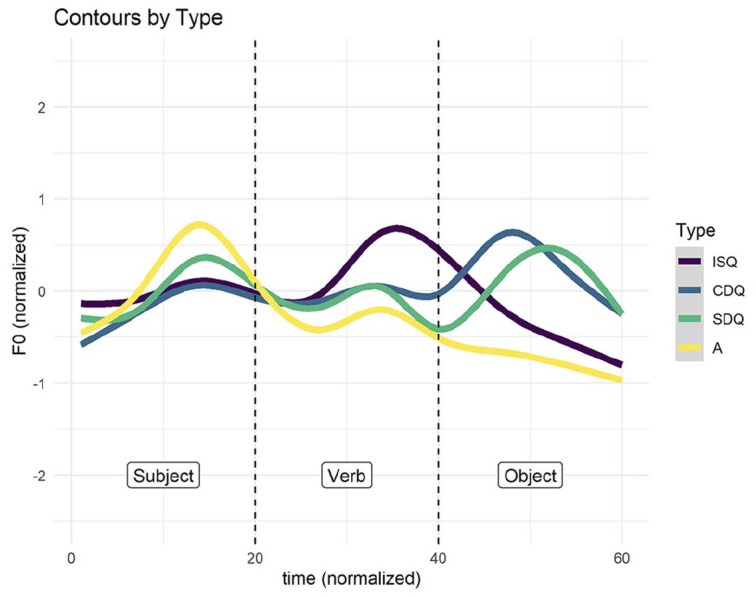
Average F0 curves for each of the four utterance types. F0, on the *y*-axis, was normalized by speaker. Time, on the x-axis, was normalized such that each of the three words was represented by 20 measurements.

As discussed at the beginning of the section, the pitch contour of every target sentence production was sampled at 58 time-points, 20 for each of the three words, with 2 measurements overlapping. The mean pitch curves for the four utterance types can be seen in Figure 7. Assertions tended to have a peak on the subject and a decline thereafter. ISQs had a peak on the verb and a decline thereafter. The two declarative question types were the most similar in terms of pitch contour, with a peak on the object for both. Surprise DQs had an additional smaller peak on the subject as well as a trough at the verb-object boundary.

Working with vectors of such size poses several challenges. First, it is unclear how to incorporate so many dependent variables into a statistical analysis. Second, because pitch tends to change gradually during the course of an utterance, a pair of proximate measurements is more likely to be similar than a pair of distal measurements. In other words, the individual measurements in the vectors are not independent of one another.

To address these two issues, principal component analysis (hence PCA) was employed as a dimensionality reduction technique. PCA has been previously employed in the literature to analyze pitch contours (Aston et al., 2010; Cvejic et al., 2010; Hadjipantelis et al., 2012), as well as phonetic phenomena more generally (Kochetov et al., 2018; Stone et al., 1997), and F(unctional) PCA has been applied to analyze the pitch of biased questions (Lo & Kiss, 2020).^9^ PCA is an unsupervised machine learning algorithm that projects the data onto a set of new and uncorrelated dimensions. The algorithm begins by finding the dimension (i.e., principal component) of the highest variance in the data. This initial dimension may be “diagonal” to the original measurements, that is, it may comprise multiple different measurements scaled by corresponding coefficients. Thereafter, PCA iteratively identifies dimensions of the highest variance (principal components) orthogonal to all previously identified dimensions. Each new dimension is, therefore, uncorrelated with all previous dimensions. The total number of principal components is the same as the number of original measurements.

More technically, for each principal component, the PCA analysis outputs a series of coefficients, the linear combination of which defines the principal component. Each coefficient corresponds to the contribution of an original measurement the principal component score. For example, a coefficient of 1 for some measurement in a principal component means that, when calculating the score for the component, the measurement is simply added to the total; a coefficient of .5 means that the measurement is scaled by a factor of .5 and added; a coefficient of −2 means that the measurement is scaled by a factor of 2 and subtracted.

The original data can be restated in terms of the principal components. Thus, each item, instead of comprising *n* measurements, can be restated as *n* principal component scores. Each principal component score describes the position of the item on the corresponding dimension of variance. For example, a score of 3 in a principal component indicates that the item has a value of 3 along some dimension of variance found in the original data as defined by the coefficients of the principal components. This score means little in isolation but may be compared to scores in the principal component for other items to see which items cluster together.

For a statistical analysis, the benefit of PCA is twofold. First, the principal component scores of the original dataset are uncorrelated. Second, the principal components are automatically ranked by the proportion of variance they account for in the original dataset. These two benefits flow naturally from the definition of the PCA: the first principal component is the linear combination of measurements accounting for the highest proportion of variance between items, and the second principal component is the linear combination of measurements accounting for the highest proportion of the remaining variance, and so on. As a result, it is often the case that the first few principal components describe most of the variance found in the original data, whereas the remaining components account for very little variance. As such, the analysis can afford to trim most of the principal components with little loss of crucial information.

In addition to facilitating a statistical analysis, PCA has the theoretical advantage of highlighting dimensions of variance in the data not obvious on the surface. PCA is, therefore, perfectly suited for domains such as intonation, where the independent variable affects different dependent variables in a different, potentially non-linear, fashion. For example, PCA can isolate the effect of a certain pragmatic property on the pitch contour, even if the pragmatic property induces a trough in one portion of the utterance and a peak in another. In effect, PCA provides a language for talking about underlying forces in the data separately from their surface correlates.

**Figure 8.**
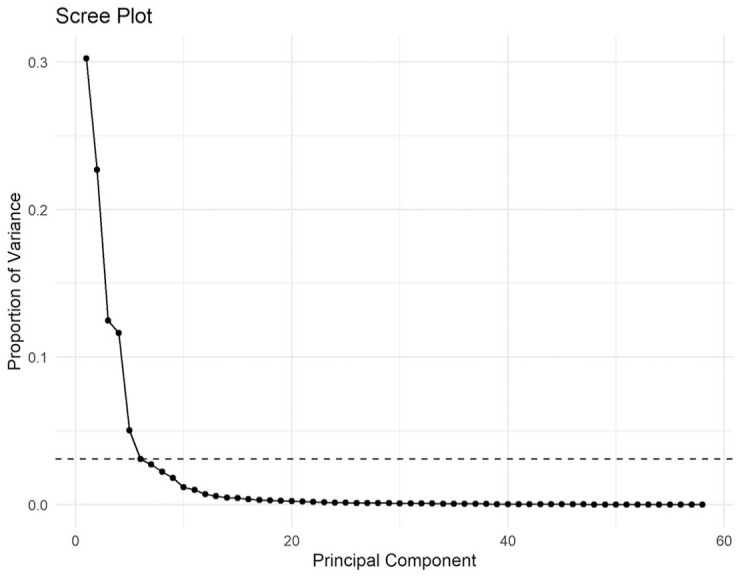
A scree plot of the PCA analysis. Principal components are on the x-axis; the proportion of variance accounted for by each principal component is on the y-axis. Only the first six principal components (dashed line for cut-off) were used in the analysis.

Figure 8, known as a scree plot, illustrates the proportion of the total variance in the data accounted for by each principal component in the analysis of the production experiment data. As can be seen in the figure, the first six principal components account for 85.2% of the total variance. This was deemed to be sufficient, and the remaining 52 components were removed from the analysis. As a result, each pitch curve was represented with only 6 values at the cost of losing 14.8% of the original variance.

**Figure 9.**
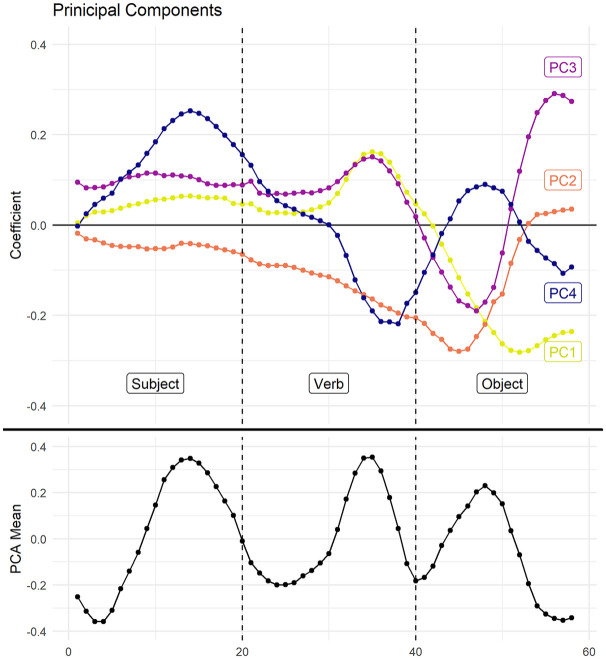
The coefficients of the first four principal components. Time (normalized) is on the *x*-axis.

Figure 9 plots the coefficients for the first four principal components, as well as the mean F0 values (black), for the 58 F0 sampling time steps in the utterance. The coefficient corresponds to the linear contribution of the F0 measurement at the particular time step to the principal component score. A positive coefficient at a time step indicates that the principal component score increases in proportion to the coefficient multiplied by the normalized pitch measurement at that step. A negative coefficient at a given time step indicates that the principal component score decreases in proportion to the coefficient multiplied by the normalized pitch measurement at that step. For example, it can be seen in Figure 9 that a high pitch at around measurement 50, that is, about half-way through the object of the utterance, decreases the score for the first principal component (PC1) but increases the score for the fourth principal component (PC4).

Generally speaking, utterances with pitch curves visually resembling the coefficient curve of the principal component exhibit high scores in the principal component, since F0 peaks aligned with positive coefficients and F0 troughs aligned with negative coefficients both contribute positively to the principal component score. Therefore, some general trends can be discerned from the coefficients in Figure 9 alone. For example, with the exception of PC4, which exhibits a peak on the subject, the difference in the coefficients of the principal components is most pronounced at the end of the utterance, the end of the verb and throughout the object, where the principal components deviate from 0 the most. This asymmetry indicates that most of the prosody distinguishing the utterances in the dataset is contained in the second half of the utterance. In other words, utterances with different F0 contours tend to be different in the second half rather than the first.

A difference between utterance types could appear in any of the principal components. As such, the statistical analyses for each of the six principal components were conducted separately and mirrored the analysis performed for duration in Section 4.3.1. For each of the principal components, a mixed-effects model was run using the *lme4* package (Bates et al., 2015) in *R* (R Core Team, 2019). The principal component score of the utterance was the dependent variable predicted by the model. Utterance type (reference level: ISQs) was set as the independent variable. Participant and item were included as random effects. By-utterance type slope per participant was also included in the model. Thus, the formula for the six mixed-effects model can be seen in (22), where *i* refers to the index of the principal component (1–6).

(22) PC_*i*_-score ~ type + (type|participant) + (1|item)

**Figure 10.**
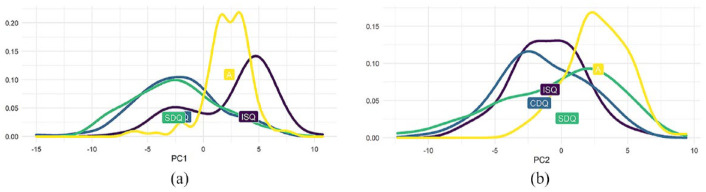
PC1 and PC2. (a) PC1: ISQ, A > CDQ, SDQ (b) PC2: A > ISQ, CDQ, SDQ.

**Figure 11.**
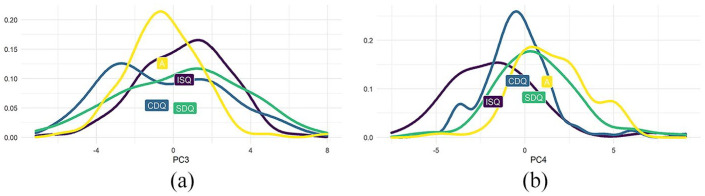
PC3 and PC4. (a) PC3: ISQ > CDQ, SDQ, A (b) PC4: A, SDQ > ISQ, CDQ.

**Figure 12.**
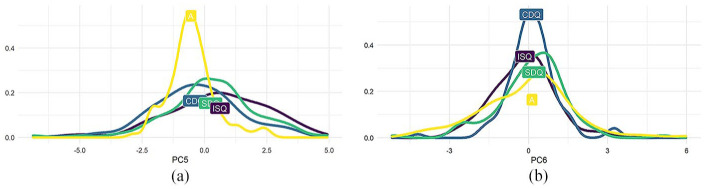
PC5 and PC6. (a) PC5: ISQ, SDQ > CDQ, A (b) PC6: N/A.

To see if the principal component differentiated between each utterance-type pair, a post hoc test was run on the model interactions using the *phia* package (De Rosario Martinez et al., 2015) in R. Utterance type was set as the pairwise comparison. The results for the mixed-effects models as well as the post hoc interaction test can be seen in Tables 7–9, where the *p*-values are adjusted using the Holm method. Meanwhile, Figures 10–12 present the distribution of utterances along each of the six principal components.

**Table 7.** Model output: PC1 (Left) and PC2 (Right).

	Estimate		*SE*	*t*		Estimate		*SE*	*t*
(Intercept)	2.1828		0.4494	4.858	(Intercept)	2.6874		0.4117	6.528
Utterance type CDQ	−4.4132		0.4587	−9.621	Utterance type CDQ	−4.0638		0.5969	−6.808
Utterance type ISQ	0.3229		0.5016	0.644	Utterance type ISQ	−3.7173		0.5257	−7.072
Utterance type SDQ	−4.6616		0.6158	−7.57	Utterance type SDQ	−2.9634		0.6518	−4.547
Post hoc tests for PC1					Post hoc tests for PC2				
	Value	df	χ^2^	Pr(> χ^2^)		Value	df	χ^2^	Pr(> χ^2^)
A−CDQ	4.4132	1	92.5605	< 2.2e−16***	A−CDQ	4.0638	1	46.3434	4.96e−11***
A−ISQ	−0.3229	1	0.4143	1	A−ISQ	3.7173	1	50.0092	9.18e−12***
A−SDQ	4.6616	1	57.3106	1.49e−13***	A−SDQ	2.9634	1	20.6714	2.18e−05***
CDQ−ISQ	−4.7361	1	83.675	< 2.2e−16***	CDQ−ISQ	−0.3465	1	0.4561	0.6238
CDQ−SDQ	0.2483	1	0.1987	1	CDQ−SDQ	−1.1004	1	3.2887	0.2093
ISQ−SDQ	4.9844	1	44.2024	8.883−11***	ISQ−SDQ	−0.7539	1	1.0225	6.24e−01

*** *p* < 0.001.

**Table 8.** Model output: PC3 (Left) and PC4 (Right).

	Estimate		*SE*	t		Estimate		*SE*	*t*
(Intercept)	−0.50441		0.26314	−1.917	(Intercept)	1.4763		0.3822	3.863
Utterance type CDQ	0.02071		0.45078	0.046	Utterance type CDQ	−1.8916		0.3889	−4.864
Utterance type ISQ	1.05132		0.3407	3.086	Utterance type ISQ	−3.1952		0.3807	−8.394
Utterance type SDQ	0.95038		0.49006	1.939	Utterance type SDQ	−0.807		0.4138	−1.95
Post hoc tests for PC3					Post hoc tests for PC4				
	Value	df	χ^2^	Pr(> χ^2^)		Value	df	χ^2^	Pr(> χ^2^)
A−CDQ	−0.02071	1	0.0021	1.00e+00	A−CDQ	1.8916	1	23.6616	4.59e−06***
A−ISQ	−1.05132	1	9.5221	1.22e−02*	A−ISQ	3.1952	1	70.4587	2.82e−16***
A−SDQ	−0.95038	1	3.761	1.57e−01	A−SDQ	0.807	1	3.8029	5.12e−02.
CDQ−ISQ	−1.03061	1	6.956	0.04177*	CDQ−ISQ	1.3036	1	12.0378	0.0010426**
CDQ−SDQ	−0.92967	1	5.545	0.07413.	CDQ−SDQ	−1.0846	1	13.4229	0.0007457***
ISQ−SDQ	0.10094	1	0.0583	1.00e+00	ISQ−SDQ	−2.3882	1	27.6637	7.22e−07***

* *p* < 0.05; **p < 0.01; ***p < 0.001.

**Table 9.** Model outputs: PC5 (Left) and PC6 (Right).

	Estimate		*SE*	t		Estimate		*SE*	*t*
(Intercept)	−0.5117		0.2374	−2.155	(Intercept)	−0.08231		0.18743	−0.439
Utterance type CDQ	0.1834		0.2328	0.788	Utterance type CDQ	0.21059		0.21483	0.98
Utterance type ISQ	1.075		0.2319	4.635	Utterance type ISQ	−0.07806		0.26226	−0.298
Utterance type SDQ	0.7967		0.2787	2.859	Utterance type SDQ	0.19995		0.2342	0.854
Post hoc tests for PC5					Post hoc tests for PC6				
	Value	df	χ^2^	Pr(> χ^2^)		Value	df	χ^2^	Pr(> χ^2^)
A−CDQ	−0.18341	1	0.6206	8.09e−01	A−CDQ	−0.210593	1	0.9609	1e+00
A−ISQ	−1.07502	1	21.4824	2.143e−05***	A−ISQ	0.078065	1	0.0886	1e+00
A−SDQ	−0.79673	1	8.1749	1.69899e−02*	A−SDQ	−0.199949	1	0.7289	1e+00
CDQ−ISQ	−0.8916	1	16.0796	0.0003037***	CDQ−ISQ	0.288658	1	2.5037	0.6815
CDQ−SDQ	−0.61331	1	4.8064	0.0850602.	CDQ−SDQ	0.010644	1	0.0037	1e+00
ISQ−SDQ	0.27829	1	0.6946	8.091824e−01	ISQ−SDQ	−0.278014	1	2.327	6.815e−01

* *p* < 0.05; ***p < 0.001

To summarize the results, the first five principal components exhibit evidence of some significant effect of utterance type. Utterance type was not a significant predictor for PC6. Every pair of utterance types was distinguished by at least one principal component. No principal component distinguished all four utterance types.

**Figure 13.**
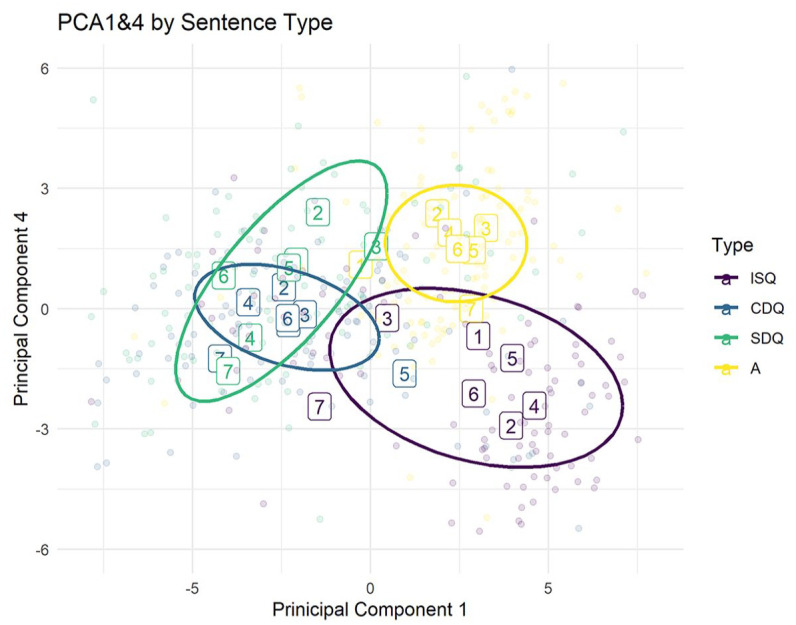
Utterances plotted by PC1 on the *x*-axis and PC4 on the *y*-axis. Intended utterance type is indicated by color. Each utterance is a single point. The labels correspond to means for each target sentence (see Table 4) in each of the four conditions. Ellipses are drawn around utterance-type regions with a .95 confidence interval using the stat_ellipse function in R.

Although five of the six principal components analyzed were correlated with utterance type, of particular interest are PC1 and PC4, as these two components alone can differentiate between the four utterance types in our study.^10^ ISQs and assertions are high in PC1, whereas confirmative and surprise DQs are low in PC1. Assertions and surprise DQs are high in PC4, whereas ISQs and confirmative DQs are low in PC4. As can be seen in Figure 13, the utterance types are distributed relatively evenly in these two dimensions. PC4 is of note here, as it is the only principal component to distinguish confirmative DQs from surprise DQs. The first three components account for a greater proportion of between-item variance but do not display any significant difference between the two declarative question types.

**Figure 14.**
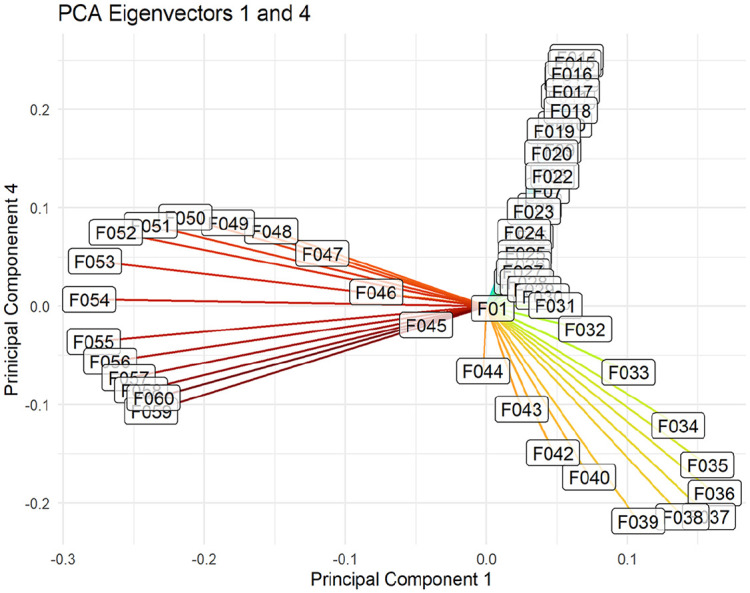
Eigenvectors for the 60 pitch measurements plotted in PC1 and PC4. Each label corresponds to the contribution of a high pitch measurement at that point to the utterance position in a PC1 ~ PC4 plot, as in Figure 13.

To make sense of the phonetic interpretation of the principal components, it can be useful to plot the coefficients in an eigen-plot. The eigen-plot in Figure 14 displays the coefficients for PC1 and PC4, two principal components that contrast the four utterance types in the dataset. Each label in the eigen-plot corresponds to the time step of the F0 measurement. The position of the label corresponds to PC1 and PC4 coefficients for F0 measurements at that time step. For example, F0 measurements at F050 (half-way through the object) have a low coefficient in PC1 and a high coefficient in PC4, meaning that high pitch values at this time step contribute negatively to the PC1 score and positively to the PC4 score, while low pitch measurements contribute positively to the PC1 score and negatively to the PC4 score. Recall that each word in the target phrase contributed the same number of measurements. Thus, measurements F01–F020 were taken from the subject, measurements F021–F040 were taken from the verb, and measurements F041–F060 were taken from the object.

Observe that the coefficients F035–F040 in Figure 14, corresponding to an increase in pitch at the tail end of the verb, are in the bottom right corner, high in PC1 but low in PC4. Observe also that utterances in the bottom right corner of Figure 13, high in PC1 but low in PC4, are predominately in the ISQ condition. In other words, a high pitch at the tail end of the verb appears to be a good predictor that an utterance is high in PC1 and low in PC4, which in turn is an indicator that the utterance is an ISQ. Likewise, the eigenvectors for measurements F014–F020 in Figure 14, corresponding to an intonation peak at the tail end of the subject, indicate that the utterance is high in PC4 (i.e., an assertion or a surprise declarative question). Finally, the eigenvectors for F050–F060 in Figure 14, corresponding to an intonation peak at the very end of the utterance, indicate that the utterance is low in PC1 (i.e., one of the two declarative question types). Note that coefficients for F0 measurements at adjacent time steps tend to be adjacent in the eigen-plot because pitch changes gradually during the course of the utterance.

### 4.4 Discussion

We use the eigenvectors for pitch measurements in PC1–PC4 space to gain a rough understanding of what the pitch contour for each utterance type is like. It should be noted that PCA is a data-driven machine learning algorithm, and its principal components are simply dimensions of the highest variance in the data. As such, important prosodic features may be missed if they do not serve to differentiate the utterances in our data set. Nevertheless, the PCA analysis proves useful in speculating about some of the prosodic properties of utterance types.

**Figure 15.**
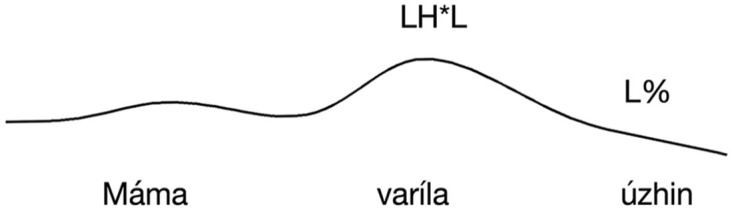
The mean pitch contour of an information-seeking question.

**Figure 16.**
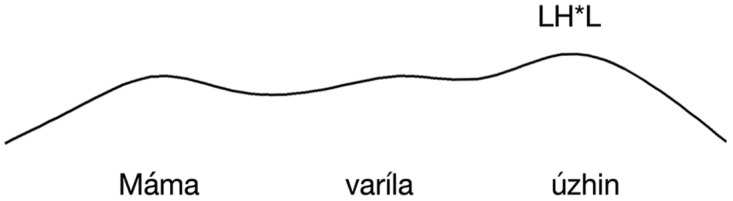
The mean pitch contour of a confirmative declarative question.

**Figure 17.**
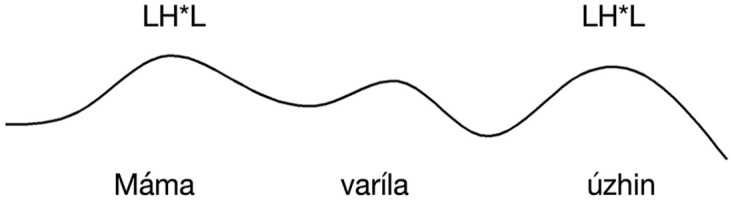
The mean pitch contour of a surprise declarative question.

**Figure 18.**
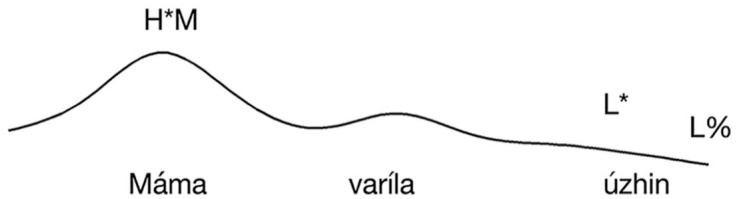
The mean pitch contour of an assertion.

In accordance with eigenvectors for measurements 50–60 in Figure 11, for utterances low in PC1 (DQs), we assume a high-pitch accent on the object with a gradual decline. In contrast, for utterances high in PC1 (assertions and ISQs), we assume a L% boundary tone. In accordance with eigenvectors for measurements 9–19, for utterances high in PC4 (assertions and surprise DQs), we assume a pitch accent on the subject with a gradual decline. Finally, in accordance with eigenvectors for measurements 33–43, for utterances low in PC4 and high in PC1 (ISQs), we posit an additional high-pitch accent on the verb with a steep decline. The mean intonation contours for the four utterance types are shown in Figures 15–18.

Transcriptions were made to align with the ToRI standard as proposed by Odé (2008a, 2008b). The pitch accent of ISQs, Bryzgunova’s IC-3, corresponds in ToRI to LH*L (Igarashi, 2006; Odé, 2008a) (or to LH*, see Odé, 2008b, and Kachkovskaia et al., 2020), and is followed by a low boundary tone L%.

DQs, too, have a rise-fall contour similar to that of ISQs, but on the last word instead of the verb, which Odé (2008a) describes as LH*L. Our measurements support this analysis, and we conclude that the LH*L contours on the three question types considered here all represent Bryzgunova’s (1977) IC-3. Our measurements show that the posttonic part of the LH*L accent in DQs is somewhat higher than in ISQs, however, this may be due to the fact that the contour is realized on the last word. Even though the posttonic low tone falls on the last syllable and could therefore in principle be fully realized, in practice, there may not be enough time for that and the function of an LH*L contour is still maintained even if the posttonic L does not fall as low as in ISQs. A perception experiment could verify or falsify this hypothesis, given that the LH* and LH*L contours, which are both pronounced as LH* when they occur on a single syllable, have been perceptually distinguished by listeners when they fell on the last syllable of the utterance (Odé, 2005).

Surprise DQs and assertions bear a high tone on the stressed syllable of the subject. While in the case of surprise DQs, this contour is a rise-fall (LH*L), assertions do not involve a sudden rise on that syllable, which is characteristic of questions. Even though in previous work the peak of the intonation contour of ISQs has been shown to be produced higher than the peak of the intonation contour of assertions or that of wh-questions (Igarashi, 2006), it is reasonable to label the peak of assertions as H* based on our measurements. The posttonic part does not clearly involve a fall to the low register of the speaker; we therefore mark the pitch accent on the subject as H*M.

Finally, we also assume a low boundary tone at the end of the sentence for both ISQs and assertions, as has been found previously (Bryzgunova, 1977; Odé, 2008b; Timberlake, 2004).

The PCA performed on the dataset demonstrates that two principal components, PC1 and PC4, are sufficient for a full contrast between the four utterance types of interest. PC1 appears to be sensitive to whether the utterance type involves a change in the speaker’s beliefs. PC1 is high in assertions and ISQs, which do not involve a belief change due to some contextual evidence, and it is low in DQs, which do signal such a change. The proposed correlation between PC1 and belief change is marked in Table 10 in light and dark pink shading. Given that low PC1 values correspond to high-pitch measurements in the 50–60 range (of 60), we propose that belief change is indicated by a high-pitch accent on the object in Russian SVO sentences.

**Table 10.** Speaker commitment expressed by information-seeking questions (ISQs), confirmative and surprise declarative questions (DQs), and assertions.

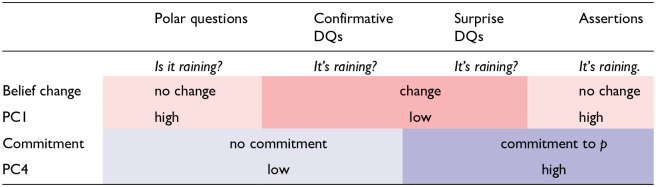

The sentence radical “it is raining” is marked as *p*.

A similar argument can be made with respect to PC4. PC4 is low in ISQs and confirmative DQs, but high in surprise DQs and assertions. PC4 thus appears to be sensitive to whether the utterance commits the speaker to *p* or not. The proposed correlation between PC4 and speaker commitment is marked in Table 10 in light and dark blue shading. Given that low PC4 values correspond to high-pitch measurements in the F09–F19 range, we conclude that a commitment to *p* is indicated by a high pitch accent on the subject in Russian SVO sentences.

All in all, the initial predictions of the production experiment were borne out: DQs exhibited a rise-fall contour on the object, and the two subtypes were prosodically distinguished in production. The latter finding is in line with the growing body of literature on the prosody of biased questions where pitch has been found to play an important role in signaling the speaker’s stance (Asu et al., 2023; Beyssade & Delais-Roussaire, 2022; Braun et al., 2018; Dehé & Braun, 2019; Dehé et al., 2018; Grice & Savino, 2003; Henriksen, 2012; Korotkova, 2023; Lo & Kiss, 2020; Mády et al., 2023; Michelas et al., 2013; Prieto & Borràs-Comes, 2018; Rett & Sturman, 2021; Vanrell et al., 2012, 2017; a.o.).

In addition to the importance of pitch in marking biased questions, this study also shows that intonational marking in biased questions is not restricted to the end of the utterance. Surprise DQs differ from confirmative ones by an utterance-initial cue, namely a peak on the stressed syllable of the subject. This finding is thus in line with previous reports on non-utterance-final intonational phenomena in biased questions (Braun et al., 2018; Dehé & Braun, 2020; Lo & Kiss, 2020; Patience et al., 2018; Petrone & Niebuhr, 2014). The proposed form-meaning relations are shown in Table 10.

As for duration, surprise DQs appeared to be significantly longer than confirmative DQs, as well as assertions and ISQs. Assertions were also found to be longer than ISQs and confirmative DQs. This, too, may be an indication that a commitment to *p* is also correlated with longer utterance duration. As mentioned in Section 4.3.1, longer duration has been found to consistently cooccur with the expression of commitment cross-linguistically and across biased question types (Braun et al., 2018; Dehé & Braun, 2019; Dehé et al., 2018; Lo et al., 2019; Lo & Kiss, 2020). We leave it to future research to explore this correlation further.

The prosodic difference between Russian confirmative and surprise DQs is a novel finding, which bears importance beyond Russian DQs. Theoretically, however, the fact that a change in the speaker’s epistemic stance is something that is marked by the form of an utterance is not surprising, given that speakers pay a lot of attention to who commits to what (see Geurts, 2019, according to whom communication is ultimately about commitment sharing). The fact that the two subtypes of DQs differ prosodically provides further support for the claim that prosody is used by speakers to signal epistemic stance. As such, prosody should be included in the semantic analysis of utterances where subtle pragmatic meaning plays an important role, as in the case of biased questions (Gussenhoven, 2004; Prieto, 2015).

The suggestion that belief change and commitment are correlated with particular prosodic properties, PC1 and PC4, in our dataset, respectively, is also novel. Correlations of this sort, where the independent variable modifies the dependent variables in non-obvious ways, are best analyzed through a PCA. The abstraction provided by PCA allows for the discussion of forces affecting the data rather than the data itself. This is particularly useful in domains where these principal components may have an independent interpretation, such as intonation with various expressions of epistemic stance or any other prosodically salient feature.

We leave several questions open. Our paper looked at SVO sentences without a narrow focus on any of the constituents. Since IC-3 can also mark focus (Bailyn, 2012), it raises the question of whether we find any differences between the object of a declarative question and the focused object of a declarative sentence in terms of how this rise-fall contour is realized. We did not discuss whether and how affective stance (i.e., emotions) interact with the prosodic cues of DQs investigated here, pitch and duration. This question is particularly relevant for surprised DQs, because even though surprise is a phenomenon that is epistemic in nature, it is natural to also express positive or negative emotions regarding the surprising fact. Relatedly, facial and manual gestures and their potential effect on the realization of the relevant contours has not been considered. While all of these issues deserve attention, we leave them for future research.

## 5 Conclusion

This study explored the interaction of declarative question type and intonation in Russian. The results of the production experiment revealed that pitch contour differed significantly in string-identical sentences produced in different contexts. Assertions exhibited a peak on the subject of the SVO string; ISQs exhibited a peak on the verb; both declarative question types exhibited a peak on the object; and surprise DQs exhibited an additional peak on the subject, and were also found to be longer than other utterance types. Assertions were found to be longer than ISQs and confirmative DQs. The four utterance types were differentiated by intonation contour alone.

A PCA of the production data showed that two principal components are enough to differentiate the four utterance types. The principal components capture semantic properties of an utterance that are independently justified in the literature, namely belief change and speaker commitment. PCA allows for the statistical analysis of high-dimensional and correlated data such as intonation contours, while also isolating dimensions of variance that may have an independent interpretation.

This study was meant to bridge the fields of semantics and phonetics and explore an understudied prosodic phenomenon in Russian. As such, it contributes to the growing body of literature that explores the form of DQs and other biased question types, where pitch and/or duration have been found to be important prosodic cues (Asu et al., 2023; Beyssade & Delais-Roussaire, 2022; Braun et al., 2018; Celle & Pélissier, 2022; Dehé & Braun, 2019; Dehé et al., 2018; Grice & Savino, 2003; Henriksen, 2012; Kiss, 2024; Korotkova, 2023; Lo & Kiss, 2020; Mády et al., 2023; Michelas et al., 2013; Prieto & Borràs-Comes, 2018; Rett & Sturman, 2021; Vanrell et al., 2012, 2017; a.o.). We suggest that the use of PCA to isolate semantic features in prosody may prove useful for other languages and for other linguistic phenomena, most importantly in the realm of biased questions. These findings contribute to the understanding of intonation and its connection to utterance type in general, and they may be useful to the study of Russian prosody in particular.

## Notes

1. By the “felicitous use” of an utterance, we mean that it embodies a plausible or natural move at that given stage of the conversation.

2. Though information-seeking and declarative questions are assumed to have the same denotation, these denotations are derived differently. For the details of the semantic analysis, see Farkas and Roelofsen (2017); for alternative analyses, see Malamud and Stephenson (2015) or Rudin (2022). This question does not affect the main claims of the paper.

3. See Poschmann (2008), though, who treats bias as partial commitment.

4. See also Odé’s (1989) (re)classification of Russian pitch movements.

5. According to Gutzmann et al. (2020), what appears to be a verum focus intonation in information-seeking polar questions is in fact the lexical operator verum. We leave the issue of whether IC-3 contributes verum or focus for future research.

6. See Makarova (2001, 2007) for further experiments on distinguishing short declarative, interrogative, and exclamative sentences in Russian.

7. Note that for (19a) to be grammatical with an NPI, the verb has to be in the imperfective aspect.

8. Note that Makarova’s original terms are “neutral (unemotional) and surprised (emotional) yes/no questions.”

9. Though FPCA, too, is a dimensionality reduction technique, it differs in many respects from PCA (see Gubian et al., 2015).

10. PC4 and PC5 together can also distinguish all four utterance types but account for a smaller proportion of the total variance. No other combination of two principal components was sufficient for a full contrast.
